# Biophysical and biochemical changes in skin health of healthcare professionals using respirators during COVID‐19 pandemic

**DOI:** 10.1111/srt.13239

**Published:** 2022-11-16

**Authors:** Nkemjika Abiakam, Hemalatha Jayabal, Kay Mitchell, Dan Bader, Peter Worsley

**Affiliations:** ^1^ School of Health Sciences University of Southampton Southampton UK; ^2^ Critical Care Team University Hospital Southampton Foundation Trust Southampton UK

**Keywords:** COVID‐19, inflammatory biomarkers, personal protective equipment (PPE), respirator protective equipment (RPE), skin health

## Abstract

**Background:**

Personal protective equipment, including respirator devices, has been used to protect healthcare workers (HCWs) during the COVID‐19 pandemic. These are fitted to skin sites on the face to prevent airborne transmission but have resulted in reports of discomfort and adverse skin reactions from their continued usage. The present study addresses the objective changes in both the structural integrity and biological response of the skin following prolonged and consecutive use of respirators.

**Materials and methods:**

A longitudinal cohort study, involving 17 HCWs who wear respirators daily, was designed. Changes in the barrier properties and biological response of the skin were assessed at three facial anatomical sites, namely, the nasal bridge, left cheek and at a location outside the perimeter of respirator. Assessments were made on three different sessions corresponding to the first, second and third consecutive days of mask usage. Skin parameters included transepidermal water loss (TEWL), stratum corneum (SC) hydration and erythema, as well as cytokine biomarkers sampled from sebum using a commercial tape.

**Results:**

The cheek and the site outside the perimeter covered by the respirator presented minimal changes in skin parameters. By contrast, significant increases in both the TEWL (up to 4.8 fold) and SC hydration (up to 2.7 fold) were detected at the nasal bridge on the second consecutive day of respirator‐wearing. There was a high degree of variation in the individual expression of pro‐and anti‐inflammatory cytokines. Increasing trends in nasal bridge TEWL values were associated with the body mass index (*p* < 0.05).

**Conclusions:**

The most sensitive objective parameter in detecting changes in the skin barrier proved to be the increase in TEWL at the nasal bridge, particularly on the second day of consecutive respirator usage. By contrast, other measures of skin were less able to detect remarkable variations in the barrier integrity. Consideration for protecting skin health is required for frontline workers, who continue to wear respirators for prolonged periods over consecutive days during the pandemic.

## 1 INTRODUCTION

With the worldwide surge of the coronavirus pandemic, healthcare workers (HCWs) have been mandated to use respiratory protective equipment (RPE) to protect them from airborne COVID‐19 particles. These typically involve disposable respirators ranging from a filtration level of 95% to 99%, which are tightly fitted to the face. If fitted correctly, they form an airtight seal around the mouth and nose. Respirators are manufactured according to generic designs and incorporate stiff materials, which can create a mismatch in both the underlying geometry of the face and stiffness of the skin and soft tissues.^1^ Due to the tight‐fitting nature of these devices and their design principles, prolonged use could lead to changes in skin health, with a range of adverse reactions reported by HCWs.^2^ These skin responses can be a direct result of the elevated pressure and frictional forces applied to the skin interface,^3^ as well as the creation of an occlusive microenvironment, resulting in the accumulation of heat and humidity.^4^

Various non‐invasive bioengineering tools are available to objectively monitor changes in skin health following external challenges.^5^ These include physical sensors to monitor the mechanical and thermal skin contact conditions, biophysical parameters to evaluate barrier function and hydration, and biomarkers reflecting local skin physiology including inflammation and ischemia.^6^ Indeed, parameters such as transepidermal water loss (TEWL), stratum corneum (SC) hydration, skin erythema and surface pH have been used in studies following skin exposure to mechanical loading,^7^ moisture^8^ and chemical irritation.^9^ Recently, one randomized crossover study, evaluating N95 and surgical masks, reported increased levels of TEWL, SC hydration and pH following N95 respirator application.^10^ However, the study only involved a cohort of 20 healthy volunteers, who applied the masks for a maximum period of 4 h, without performing any functional activities. In a similar study design, Peko et al.^4^ measured facial contact forces, skin temperatures and sub‐epidermal moisture to evaluate the effects of surgical facemasks and N95 respirators on healthy volunteers. Their findings demonstrated that the respirators, which require a tight seal, created high contact forces and thermal loads at the skin interface compared to standard surgical facemasks. However, both studies do not reflect the clinical scenario, where staff can apply the masks for in excess of 8 h in a given working shift, performing various activities during their routine daily working. In those studies, which included HCW participants, respirator application has been restricted to 2 h. However, this was shown to be sufficient to create a statistically significant increase in TEWL, erythema and temperature.^11^, ^12^

To date, no studies have utilised biochemical markers, which reflect the health status of the skin.^6^ In particular, inflammatory markers sampled non‐invasively from the sebum released at the skin surface, have been previously employed as early indicators of skin damage as a consequence of pressure and shear forces.^13^, ^14^, ^15^ This approach has enabled the quantification of a variety of pro‐and anti‐inflammatory cytokines. Indeed, up‐regulation of specific proteins including Interleukin‐1 alpha (IL‐1α) and the receptor antagonist (RA), Interleukin‐1 receptor antagonist (IL‐1RA), were reported in a study evaluating the effects of strap tension during non‐invasive ventilation mask application.^16^ Similar increases were also detected when the skin was subjected to either pressure alone or in combination with occlusion.^8^ Given the similar nature of the respirators to the occlusive device's studies to date, it is hypothesised that a similar upregulation of inflammatory biomarkers may be present following prolonged RPE application.

To date, research has focussed on respirator application on healthy volunteers and HCWs for short application periods, with parameters of skin health limited to biophysical assessments. There is a need for more empirical data pertaining to the skin responses to repeated application of respirators, as observed in a typical working week for HCWs. Accordingly, the present study aimed to investigate the biophysical and biomarker changes in skin health following RPE application using a longitudinal design over repeated clinical shifts in HCWs.

## 2 MATERIALS AND METHOD

### 2.1 Participants

HCWs were recruited from COVID‐19 high‐risk departments of one UK University Hospital healthcare provider via poster advertisement and gatekeeper communication. Inclusion criteria consisted of individuals over 18 years of age, who employ FFP2/3 masks on a daily basis while attending to clinical commitments, who worked a minimum of three consecutive clinical shifts per week. Exclusion criteria included individuals with no active skin conditions at the facial sites of investigation, allergies or sensitivity to adhesive tape and the inability to attend a minimum of two out of the three assessment sessions. The study was approved by the UK Health Research Authority committee (IRAS 285764), and written informed consent was obtained from participants prior to commencing the study.

### 2.2 Study protocol

**FIGURE 1:**
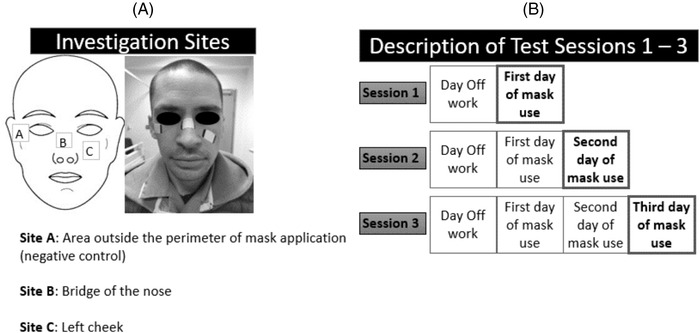
(A) Investigation sites associated with a control site ‘A’, and sites under the respirator mask, namely the bridge of the nose ‘B’ and left cheek ‘C’. (B) Study protocol including each test session and the corresponding repeated days of respirator application

The study was conducted during the second wave of the COVID‐19 pandemic in the UK (December 2020 to March 2021). Three anatomical locations on the face, namely an area outside the perimeter of respirator application (negative control denoted A), the bridge of the nose (B) and the load‐bearing area of the left cheek (C), were investigated (Figure 1A). The areas of investigation B and C were chosen due to being reported to be prone to damage following prolonged use of respirators.^17^, ^18^ Participants who agreed to take part in the study were tested on three different occasions based on a standardised protocol, as summarised in Figure 1B. Participants were requested to avoid the application of any moisturizer and/or cosmetics on the face on each of the assessment days. In addition, they were requested to use only surgical masks for protection during off‐duty days to avoid compromising skin health. During the test session, each participant acclimatized to an indoor environment, and their face dried with paper towels (Tork, Bedfordshire, UK) prior to commencing skin assessments. All test sessions were conducted in a temperature and humidity‐controlled laboratory (room temperature of 22.5 ± 0.7⁰C and relative humidity of 42 ± 6%) before and after the participant's working shift. Three distinct data collection sessions were used:

1. Session 1: participant first day of mask usage following return to work after a period of absence (minimum of 24 h)
2. Session 2: second consecutive day of mask usage in a given working week
3. Session 3: third consecutive day of mask usage in a given working week

### 2.3 Skin biophysical and biochemical assessment

At the start of the first test session, demographic and anthropometric data were collected from each participant. This include age, gender, ethnicity, height, weight, body mass index (BMI), working hours, type of respirator used, number and frequency of respirator removal, and pain perception while wearing the device.

The facial skin health of participants was assessed during each test session pre‐ and post‐respirator application, using three biophysical parameters. These included TEWL, SC hydration and erythema. TEWL was investigated using a Tewameter (TM 300, Courage & Khazaka, Germany), which incorporates an open‐chamber probe, which was gently placed in contact with the skin for 1 min during which equilibrium was achieved. SC hydration and erythema were assessed using the capacitance‐based Corneometer (CM 825, Courage & Khazaka, Germany) and the narrow‐band reflectance spectrophotometer Mexameter (MX18, Courage & Khazaka, Germany), respectively. Both devices were placed in contact with the test sites, and the mean of five repeated measurements was recorded for each parameter. The outputs of the parameters were measured in g/h/m^2^ for TEWL and arbitrary units (AUs) for both SC hydration and erythema. Participants’ skin assessments were performed generally after 10 min following the removal of RPE, which corresponded to the time taken for the individual HCW to transfer from their various hospital departments to the controlled lab setting. All measurements were performed following a specific order^19^ and according to recommended guidelines for their use.^20^

Inflammatory skin biomarkers were evaluated non‐invasively by collecting sebum from the skin surface of each participant, using commercial Sebutape patches (32 × 19 mm) (CuDerm, Dallas, TX, USA). The sebutapes were attached to the skin (Figure 1A), by means of tweezer and gloved hands, and held in place for 2 min prior to removal. Subsequently, they were placed in appropriate labelled sterile containers and stored at −80⁰C until biochemical analysis

### 2.4 Biochemical analysis

The extraction of skin inflammatory biomarkers was performed following a standardized protocol,^21^ optimised with the introduction of chemical and mechanical stimuli to improve the extraction efficiency. To review briefly, frozen Sebutape samples were thawed to room temperature, and a 0.85 ml solution of phosphate‐buffered saline (PBS) (Sigma‐Aldrich Co, St. Louis, Missouri, USA) and 0.1% dodecylmaltoside (DDM) (Thermo Fisher Scientific, UK) was added to each container. After 1 h of vigorous shaking and immersion in the solution, the containers were sonicated for 5 min, the Sebutapes discarded, and 0.5 ml of the extraction buffer was transferred into vials for centrifugation. Subsequently, the vials were centrifuged for 10 min at 15000 g, whilst being maintained at a constant temperature of 4⁰C. The supernatants were discarded and the pellets vortexed for 10 s. The samples were then processed using U‐Plex immuno‐assay kits (Meso Scale Diagnostics, USA) to quantify the concentration of inflammatory and anti‐inflammatory biomarkers (e.g., IL‐1α, tumor necrosis factor alpha (TNF‐α), Interleukin 8 (IL‐8) and IL‐1RA) over the nasal bridge (site B) of the participants.

### 2.5 Data analysis

Raw data were imported into Excel (Microsoft office package 2019, USA) for analysis. Values from TEWL, SC hydration, erythema and inflammatory biomarker concentrations were normalised to the baseline for each test session (post/pre‐respirator application ratio) in order to enable comparisons of relative variations across the experimental time frame and between the participants.^8^ ^,22^ Shapiro Wilk test and D'Agostino‐Pearson were used to assess the distribution of the data. Accordingly, a parametric two‐way analysis of variance with replication was employed to evaluate the effect of repeated respirator application derived from each test session and the difference between measurement sites for each parameter. Paired samples *t*‐Test was used to compare measurements taken between specific test sessions. Cluster analysis was conducted for normalised inflammatory biomarkers, where combined cytokine rank for each participant was collated for each test session. The integrated cytokine response was estimated by ranking the absolute values of all four cytokines for each time point and summing them across all participants in the cohort.^8^ This would result in a maximum rank sum of 204, based on the number of time points (3), participants (17) and cytokines (4). Pearson correlation coefficient was used to examine possible associations between skin health and other demographic and anthropometric data. Differences were considered to be statistically significant at the 5% level (*p* < 0.05).

## 3 RESULTS

The study recruited 17 HCWs (15 females and two males), who use RPE (FFP2 or FFP3) on a regular basis during established clinical shift patterns (Table 1). One participant contracted COVID‐19 during the study and was withdrawn for further assessment. The periods between consecutive test sessions varied for practical reasons, ranging between 1 to 8 weeks. The participants’ age ranged between 22 and 61 years (mean age 33 ± 11 years), with a mean height and weight of 1.70 ± 0.1 m and 69.7 ± 17.1 kg, respectively. The mean corresponding BMI was 25.1 ± 5.4 kg/m^2^. Participants included nurses (*n* = 8), doctors (*n* = 2) and other health‐related professions (*n* = 7). All participants were fit tested using a standardised procedure^23^ prior to employing respirators, except for the two who used the N95 device. Approximately one half of the participants (9/17) reported pain when employing RPE during clinical duties. The approximate frequency of breaks recorded by participants, as summarised in Table 1, were similar for each session of data collection, as they followed an established working pattern.

**TABLE 1:** Demographic and anthropometric data of study participants with detail of respirator use and any associated adverse reactions to the skin

ID	Profession	Gender	Ethnicity	Age (Years)	BMI (kg/m^2^)	Mask make	Working hours	Breaks from mask	Adverse reactions to respirator protective equipment (RPE)
1	Nurse	Female	White	29	20.3	Aura 1863+	12	4	Spots, dry skin
2	Nurse	Female	White	28	22.5	Aura 1863+	12	2	Itchiness, excessive sweating
3	Doctor	Female	White	41	23.1	Aura 9330+	8	2	Spots, itchiness
4	Nurse	Female	White	61	24.6	Aura 1863+	8	2	None
5	Nurse	Female	White	33	39.4	Aura 1863+	12	3	Spots, lumps
6	Other^a^	Female	White	40	34.5	Alpha Solway 3030v	10	3	None
7	Nurse	Female	White	28	22.3	Aura 9330+	12	3	Spots, itchiness
8	Other^a^	Male	White	30	23.0	N95	10	4	Excessive sweating
9	Other^a^	Female	White	22	25.1	Aura 1863+	7.5	1	Spots, dry skin
10	Other^a^	Female	Asian	26	25.0	3 M 8835+	8.5	1	Spots, dry skin, excessive sweating, headaches
11	Nurse	Female	White	28	20.3	Aura 9330+	12	2	Itchiness, spots, excessive sweating
12	Other^a^	Female	White	30	19.8	N95	8	4	None
13	Other^a^	Female	White	26	25.1	Aura 9330+	8	1	Spots
14	Nurse	Female	White	57	25.2	Aura 9330+	10	1	None
15	Other^a^	Female	White	23	33.5	Aura 1863+	12	3	Dry skin, rashes, spots, itchiness
16	Doctor	Female	Asian	35	20.2	Aura 1863+	9	4	Dry skin, spots, itchiness, rashes, excessive sweating
17	Nurse	Male	White	31	22.5	Aura 9330+	12	3	Dry skin

^a^ Other includes healthcare assistants, operations managers and clinical trials assistants who were redeployed to COVID departments.

### 3.1 TEWL

**FIGURE 2:**
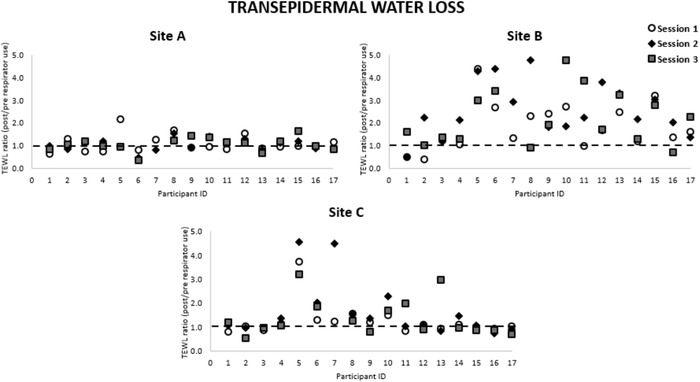
Ratio changes in transepidermal water loss (TEWL) values from pre‐ to post‐respirator application for each participant on the three test sessions at sites A, B and C

Variations in the skin barrier properties, as a function of the TEWL parameter, assessed across the test sessions are shown in Figure 2. Absolute TEWL values ranged from 5.7 to 66.9 g/h/m^2^ at the different sites across the sessions. The data revealed relatively small within‐participant variability between time points at site A (negative control), with ratio values ranging between 0.4 and 2.2. By contrast, increased ratio TEWL values were evident at site B (Figure 2), demonstrated by an increase in ratio from pre‐ to post‐respirator application (Table 2). Analysis of the ratio TEWL values revealed differences, which were statistically significant between the three test sites (*p* < 0.001), but not significant between the three test sessions (*p* > 0.05). However, on closer examination site B revealed significant differences in TEWL ratio values between sessions 1 and 2 (*p* < 0.05).

**TABLE 2:** Sensitivity analysis of the ratio changes in the three biophysical parameters at the three facial sites for each test session

		Site A % of participants according to threshold			Site B % of participants according to the threshold			Site C % of participants according to the threshold		
Parameter	Threshold (ratio change)	Session 1	Session 2	Session 3	Session 1	Session 2	Session 3	Session 1	Session 2	Session 3
Transepidermal water loss (TEWL)	< 1.0	41	41	25	12	6	12	18	18	37
	≥ 1.0	59	59	75	88	94	88	82	82	63
	≥ 1.5	18	6	13	53	82	63	18	35	31
	≥ 2.0	6	0	0	41	71	44	6	24	19
	≥ 2.5	0	0	0	29	41	38	6	12	13
	≥ 3.0	0	0	0	12	35	31	6	12	13
Stratum corneum (SC) hydration	< 1.0	12	24	25	35	24	19	18	18	31
	≥ 1.0	88	76	75	65	76	81	82	82	69
	≥ 1.5	6	12	6	35	47	25	12	18	19
	≥ 2.0	0	0	0	6	18	19	6	0	0
	≥ 2.5	0	0	0	6	6	6	0	0	0
	≥ 3.0	0	0	0	0	0	6	0	0	0
Erythema	< 1.0	18	24	12	35	47	37	12	0	12
	≥ 1.0	82	76	88	65	53	63	88	100	88
	≥ 1.5	0	0	0	24	0	6	6	0	0
	≥ 2.0	0	0	0	6	0	0	0	0	0
	≥ 2.5	0	0	0	0	0	0	0	0	0
	≥ 3.0	0	0	0	0	0	0	0	0	0

There was a high degree of variation in the response at the nasal bridge (Site B), with a sub‐group of participants (#5, #6, #10, #13, #15) presenting with consistently high TEWL ratios following respirator application in each of the three sessions. By contrast, some participants (#1, #2, #3, #4, #14, #16) demonstrated correspondingly lower TEWL ratios (≤2.2 fold). Data revealed Site C demonstrated more consistent TEWL values, only five participants (#5, #6, #7, #10, #11 and #13) demonstrating any increases in the TEWL ratios.

### 3.2 Sensitivity analysis of biophysical skin parameters

A sensitivity analysis was performed for each parameter associated with TEWL, SC hydration and erythema using arbitrary thresholds in the pre‐ to post‐measurement ratios ranging from 1.0 to 3.0. Table 2 highlights the percentage of participants whose responses exceeded these thresholds at the different test sites. It confirms that the proportion of participants remained high with increasing thresholds for TEWL, particularly for the nasal bridge test site (B) for each of the test sessions. By contrast, with SC hydration and erythema, there was a marked reduction of these percentage values when the threshold was set above 1.0.

### 3.3 SC hydration

With respect to the SC hydration, a high degree of inter‐participant variation was observed at sites A and C, with participants displaying absolute values, which ranged between 10.3 and 95.1 AUs across the test sessions. Some changes in SC hydration following respirator usage were observed for a sub‐group of participants at site B (Table 2). As an example, participant #6 presented with elevated skin hydration values (two‐fold change), which remained consistent throughout the test sessions. In addition, a sub‐cohort of participants (#1, #5, #9, #10, #11, #15 and #17) demonstrated increased SC hydration at session 2, equivalent to ≥ 1.8 ratio change. Increase in skin hydration at session 3 was below two‐fold, except for one participant (#15) who demonstrated a four‐fold increase. Nonetheless, all the differences associated with anatomical sites and test sessions were not found to be statistically significant (*p* > 0.05 in all cases)

### 3.4 Erythema

There were few remarkable trends with relation to erythema, with participants demonstrating values, which ranged between 105.4 and 898.8 AUs at all sites across the test sessions. Indeed, for the vast majority of participants, erythema ratio changes were ≤1.5 at the anatomical sites for each of the test sessions (Table 2). The one exception involved participant #6, who presented with a 2‐ and 1.5 ratio increase at site B on sessions 1 and 3, respectively.

### 3.5 Skin cytokine response

**FIGURE 3:**
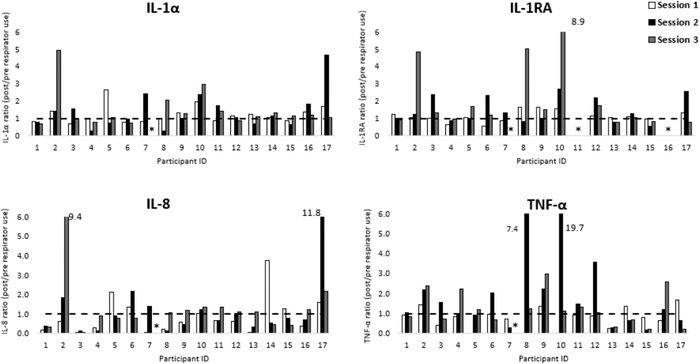
Ratio changes in four biomarkers for each participant at the nasal bridge site on the three test sessions (* indicates missing data)

Changes in skin cytokine response were evaluated in the sebum sampled at the bridge of the nose (site B). Figure 3 reveals a considerable intra‐ and inter‐inflammatory marker variability, with absolute cytokine concentrations for IL‐1α and IL‐1RA ranging from 10 to 347 pg/ml and 375 to 15591 pg/ml, respectively. The corresponding value for the low‐abundance proteins, IL‐8 and TNF‐α, ranged from 0.2 to 63.3 pg/ml and 0.3 to 12.3 pg/ml, respectively. Close examination of the data highlighted that some individuals (#2, #10, #17) presented with higher ratio changes for each of the four cytokine biomarkers. By contrast, other participants (#1, #13, #15) exhibited no changes in the ratio values for any of the cytokines and the test sessions.

Sensitivity analysis with respect to ratio change in biochemical markers are also summarised in Table 3. Differences were identified between specific biomarkers and the test sessions. For example, with IL‐1α, IL‐1RA and TNF‐α, ratios were higher in test sessions 2 and 3 compared with test session 1. By contrast, there was little difference in the IL‐8 response across the three sessions. In addition, a subset of participants demonstrated elevated biomarker responses, that is, >1.5 ratio change, for some of the cytokines. For example, this threshold ratio change was exceeded during session 2 in 35%, 33% and 41% for IL‐1α, IL‐RA and TNF‐α, respectively (Table 3).

**TABLE 3:** Sensitivity analysis of the ratio changes in four biomarkers at the three facial sites for each test session

		Site B % of participants according to the threshold		
Parameter	Threshold	Session 1	Session 2	Session 3
IL‐1α	<1.0	41	47	31
	≥1.0	59	53	69
	≥1.5	18	35	19
	≥2.0	6	18	19
	≥2.5	6	6	13
	≥3.0	0	6	6
IL‐1RA	<1.0	33	40	36
	≥1.0	67	60	64
	≥1.5	20	33	43
	≥2.0	0	33	21
	≥2.5	0	13	21
	≥3.0	0	0	21
IL‐8	<1.0	65	65	44
	≥1.0	35	35	56
	≥1.5	18	18	13
	≥2.0	12	12	13
	≥2.5	6	6	6
	≥3.0	6	6	6
TNF‐α	<1.0	76	41	44
	≥1.0	24	59	56
	≥1.5	6	41	25
	≥2.0	0	35	25
	≥2.5	0	18	13
	≥3.0	0	18	0

### 3.6 Correlational analysis with respect to intrinsic and extrinsic factors

**FIGURE 4:**
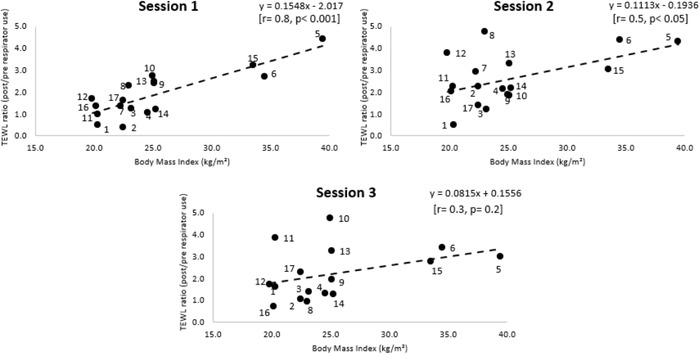
Correlations between body mass index (BMI) and transepidermal water loss (TEWL) ratio values at the nasal bridge site on the three test sessions. The data labels on the coloured dots indicate the participant ID.

The role of intrinsic factors (BMI) and extrinsic factors, that is, nature of daily respirator usage, on skin health was also investigated. TEWL at site B was selected as this represented the most sensitive skin parameter to be influenced by respirator application. The linear models revealed that there were positive correlations between BMI and TEWL ratios at the nasal bridge, as illustrated in Figure 4, which were statistically significant at sessions 1 (*p* < 0.001) and 2 (*p* < 0.05). Individual data also revealed that three participants (#5, #6, #15) in the obese BMI range (>30 kg/m^2^) presented with high TEWL values, with ratio increases ranging between 2.7 and 4.4, which were sustained throughout the three test sessions.

**FIGURE 5:**
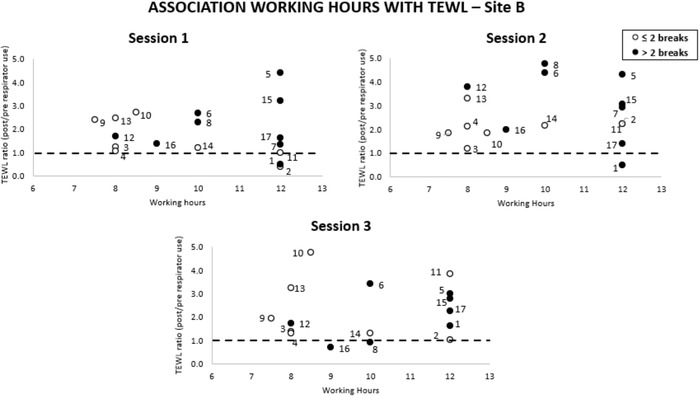
Correlations between the working hours and transepidermal water loss (TEWL) ratio values at the nasal bridge site on the three test sessions

An equivalent analysis was performed with respect to daily hours and the number of breaks during respirator application (Figure 5). Data revealed considerable variability at each session, as exemplified by participants #6, #8, #10 across the three test sessions. Neither the working hours nor the number of breaks taken during shifts yielded a significant correlation with changes in TEWL (*p* > 0.05). Indeed, some participants, that is, #2, #14 worked long shift periods (>10 h) with limited breaks and demonstrated low TEWL ratio values. By contrast, other participants, that is, #9, #10, #13, who worked an 8 h shift with a number of breaks demonstrated consistently higher TEWL ratio values.

**FIGURE 6:**
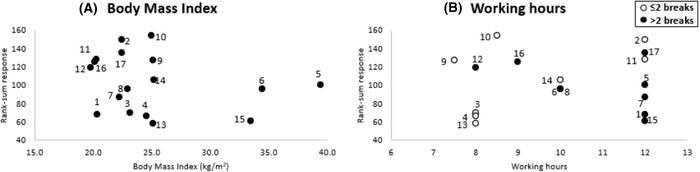
Relationship between (A) body mass index (BMI) and (B) working hours on rank‐sum response of the four biomarkers at the nasal bridge site on the three test sessions

When correlating the integrated cytokine rank‐sum with BMI (Figure 6A), the linear model was not found to be statistically significant (*p* > 0.05). The analysis also confirmed a high degree of variability in the biomarker response with respect to the duration of the working hours and the number of breaks taken within a shift (Figure 6B), with no statistically significant trend (*p* > 0.05).

## 4 DISCUSSION

One of the indirect consequences of the outbreak of the coronavirus pandemic is the adverse skin reactions as a direct result of the extensive use of personal protective equipment. Healthcare professionals are particularly affected, where the prolonged application has left them exposed to damaging mechanical and microclimate loads on the skin.^2^, ^17^, ^24^ Although the nature and frequency of these adverse reactions have been extensively reported, there is little understanding of the effects of respiratory application on the integrity of skin sites of the face. In this context, the present study demonstrated how the integration of biophysical and biochemical markers could enable a comprehensive analysis of changes in local skin physiology and barrier function. Significant changes in skin parameters have been observed within this cohort of 17 HCWs, which were particularly pronounced following two consecutive days of respirator use.

The study used an array of parameters to monitor changes in skin health in a cohort of HCWs based at one UK acute care provider. These included TEWL, SC hydration, erythema and inflammatory biomarkers sampled non‐invasively from the skin surface, during three consecutive sessions of respirator use. The consistency in TEWL and SC hydration values observed at the control site (A) for each test session provides confidence that the absolute changes at the sites in direct contact with the respirator (B and C) represented changes in skin barrier function. Indeed, the nasal bridge (site B) was most affected by the respirator application, as confirmed by the increased values in the biophysical parameters. For example, 35% of participants consistently demonstrated high TEWL values across the three test sessions (Table 2). These findings are consistent with previous studies, where a statistical increase in TEWL was reported at skin sites following 2 h^12^ and 8 h^25^ of N95 respirator application. Indeed, due to the bony prominence and cartilaginous substrate, the nasal bridge has been regularly identified as a vulnerable site for skin damage.^16^ Indeed, at this site, TEWL and SC hydration values were generally higher on the second consecutive day of mask application (session 2) with more than 70% of the participants presenting in excess of a two‐fold change from basal values in TEWL post‐respirator usage (Table 2).

By contrast, the cheek (site C) presented fewer events (∼12%), where the TEWL value was increased and revealed a few interesting trends in terms of SC hydration. This could be explained by the fact that the middle cheek and the nasolabial areas present with poor hydration compared to other facial sites.^26^ In addition, the cheek incorporates a higher proportion of soft tissues, which can provide load‐bearing capacity.^27^ Although our findings contrast with a recent study, which highlighted a significant SC hydration and TEWL increase at the cheek following the employment of respirators,^10^ these studies did not allocate breaks for respirator usage during their study design. This contrasts with the protocol adopted in the present study, where breaks were allowed (Table 1). The length of the breaks was different for each participant, ranging from 15 to 50 min, and they were part of established clinical work patterns, which were uncontrolled in the study. These breaks from respirator application could have restored the TEWL and skin hydration values toward basal (unloaded) levels, as well as influenced skin inflammatory biochemical processes.

It is of note that there were no remarkable differences observed in skin erythema, evaluated using the Mexameter device. This was surprising given that skin redness was visibly evident in the loaded sites of some participants, and other studies have reported significant evidence of redness following respirator application.^12^, ^25^ This may be due to the lack of sensitivity in measurement system^28^, ^29^ and its dependence on skin pigmentation.^7^ The application of the Mexameter was also limited by the curved location of the nasal bridge, which could have introduced errors in the detection of erythema.

Biochemical marker analysis highlighted considerable variations between participants and across the test sessions (Figure 3). While some individuals expressed consistently higher responses in the candidate biomarkers in each of the test sessions, others showed minimal up‐regulation following respirator usage. This suggests that individual sites of mechanical insult evoke a variable number of macrophages, which are responsible for the production of cytokines.^30^ In addition, the up‐regulation of IL‐1 family of cytokines, namely IL‐1α and IL‐1RA, could be a direct result of their early synthesis and storage as precursor proteins, which are released following inflammatory events. By contrast, IL‐8 and TNF alpha are mainly associated with dendritic cells and thus require the recruitment and the migration of these to the site exposed to external stimuli prior to being expressed.^31^ Accordingly, these cytokines are expressed in smaller concentrations. It is to be further noted that the interplay of pro‐ and anti‐inflammatory markers is important in the process of skin inflammation.^32^ The present study indicated equivalent up‐regulation in the pro‐inflammatory (IL‐1α) and the anti‐inflammatory RA (IL‐RA) for sessions 1 and 2 (Table 3). In session 3, however, there is a higher proportion of participants who demonstrated an up‐regulation in IL‐RA. This might indicate that more time is required in order for this cytokine to migrate and be detectable at the skin surface. The biochemical parameters have highlighted the importance of cluster analysis, where sub‐groups within a healthy cohort respond differently to given external stimuli, as has been demonstrated by previous studies from the host laboratory.^7^, ^8^

The present study has examined any correlations between intrinsic and extrinsic factors and the ratio changes in TEWL and inflammatory cytokines. As an example, the ratio changes in TEWL were significantly associated with participants’ BMI at the bridge of the nose (Figure 4). This association might be explained by the elevated perspiration generated within the occlusive micro‐environment created by the devices, as a consequence of the combined effect of high body fat mass together with the adherence of HCWs to the demanding schedules in the COVID units. Indeed, exposure to elevated moisture at the skin interface can reduce the mechanical stiffness and strength of the SC thereby increasing its susceptibility to damage.^33^ Changes in both the biophysical parameters and biochemical markers did not correlate to either the average daily working hours or the number of breaks taken by the participants during their clinical shifts (Figures 5 and 6). Indeed, the considerable variability in the responses across the cohort suggests that there are intrinsic factors coupled to other extrinsic factors, which determine the skin tolerance to load‐bearing. This contrast with findings from surveys, where HCWs subjectively reported skin reactions were associated with both the working shift time and the frequency of breaks.^2^ There were no associations between the changes in biophysical parameters and biomarkers following respirator application. This is perhaps not surprising given that each parameter was sensitive to different aspects of skin physiology and function. For example, while TEWL is used to assess the barrier properties of the skin, which is a function of the SC integrity, the inflammatory biomarkers are derived from a complex biological response of dermal and epidermal cells and tissues to the mechanical and thermal insults associated with application of the respirator.

The study cohort was limited by the relatively small HCW cohort from a single UK acute care provider. In addition, most of the participants were female from white (Caucasian) ethnicity (Table 1). There were also only small variations with respect to the age and BMI of the participants. Furthermore, the data collection was conducted over the course of three sessions, and except for the testing days, participants were allowed to use skin protective measures during their shifts. Although participants were required to avoid the application of moisturizers and/or creams, it is of note that the outputs of the parameters might be influenced by skin care behaviour on the days prior to the study assessments. In addition, due to practical reasons, data collection occurred over varying time periods (1–8 weeks), which might have impacted the nature of the skin response and in particular the inter‐subject variability in the data. Lastly due to COVID‐19 restrictions, which limited contact with the participants outside the work environment, it was not practicable to assess individual baseline during days off work.

Although it is essential to adopt RPE while working in COVID‐19 high‐risk units, strategies are required to protect skin health of heavily resourced HCWs. Indeed, what is initially seen as skin erythema and indentation marks could easily lead to skin breakdown, which could provide an access site to coronavirus, as well as other hospital‐acquired infections. Regardless of the successful fit test,^23^ HCWs still continue to report adverse skin reactions (Table 1) and discomfort while employing these devices. Therefore, healthcare organisations worldwide must acknowledge these issues and create policies to protect skin health. Collaboration with industry is required to develop new respirator designs to provide comfortable and effective respirator devices.

## 5 CONCLUSION

The current study used a multi‐array approach to assess changes in the skin health of HCWs before and after the use of RPE in routine clinical shifts. Participants varied in their response, with the nasal bridge representing the anatomical site most affected by the devices. The study demonstrated that for a sub‐group of HCWs, current respirators impair the barrier function of the skin and cause local inflammation which, if left untreated, could lead to changes in skin integrity. TEWL was the most sensitive parameter to change over the course of the longitudinal evaluation. Biochemical analysis showed an up‐regulation of IL‐1α and the RA, although considerable variability was observed, limiting comparisons between individual responses. Further studies are required to define relationships between mask designs, application periods and skin reactions.

## CONFLICT OF INTEREST

The authors have no conflict of interest.

## Data Availability

The data that support the findings of this study are available from the corresponding author upon reasonable request.
